# Enhancing the knowledge in fundus autofluorescence of optic nerve head drusen assessed with broad line fundus imaging technology

**DOI:** 10.3205/oc000222

**Published:** 2023-07-12

**Authors:** Gabriel Castilho Sandoval Barbosa, Leandro C. Zacharias, Eduardo A. Novais, Ricardo L. L. Guerra

**Affiliations:** 1 Department of Ophthalmology, Suel Abujamra Institute, São Paulo, Brazil; 2 Department of Ophthalmology, University of São Paulo, São Paulo, Brazil; 3 Department of Ophthalmology, Centro Oftalmológico Città, Rio de Janeiro, Brazil; 4 Department of Ophthalmology, Clínica de Olhos Leitão Guerra, Salvador, Brazil

**Keywords:** broad line fundus imaging, fundus autofluorescence, optic nerve head drusen

## Letter to the editor

The concept of retinal autofluorescence (AF) came to light in the 1970s when an autofluorescent signal of optic nerve head drusen (ONHD) was noted prior to the injection of fluorescein dye [1]. Nowadays, several devices offer distinct methods for acquiring short-wave fundus autofluorescence (SW-FAF) images, all of them being clinically useful in assessing the presence of lipofuscin, a naturally occurring ocular fluorophor which reflects the general health of the photoreceptor and retinal pigment epithelium (RPE). Each device provides a different image acquisition method; thus, discrepancies exist in the images obtained. For example, fundus cameras feature a high-energy white flash that streams through a wideband excitation filter and a series of mirrors and apertures to obtain images. On the other hand, confocal scanning laser ophthalmoscope (cSLO) emits a single wavelength, which will only excite fluorophores that absorb light with a peak excitation near that specific wavelength [2].

Broad line fundus image (BLFI) technique is a hybrid of both cSLO and traditional fundus photography. By utilizing line scanning illumination with light-emitting diodes (LEDs) and an aperture confocal to the illumination, this system illuminates and detects retinal images in two wavelength ranges: fundus autofluorescence (FAF)-Blue (435–500 nm) and FAF-Green (500–585 nm) [3]. Both are categorized as short-wavelength, but it is well known that green light can reach deeper layers of the retina, being absorbed less by the luteal pigments of the macula, and allowing a better definition of lesions in the underlying RPE.

ONHD are a form of calcific degeneration in some of the axons of the optic nerve that may give a swollen-looking appearance, and are usually formed early in life. ONHD are diagnosed with fundoscopic examination, but some cases may require B-scan ultrasonography, AF, and optical coherence tomography (OCT) to confirm the lesion [4]. The features of ONHD in FAF imaging are well described in many previous publications [4], [5]. Yan et al. [4] recently described the peculiarities of ONHD in FAF-Blue and -Green by using a cSLO system from two different devices. Despite a considerable amount of meaningful information, we were unable to find a description of FAF imaging in ONHD using BLFI technology in the literature.

**Figure 1:**
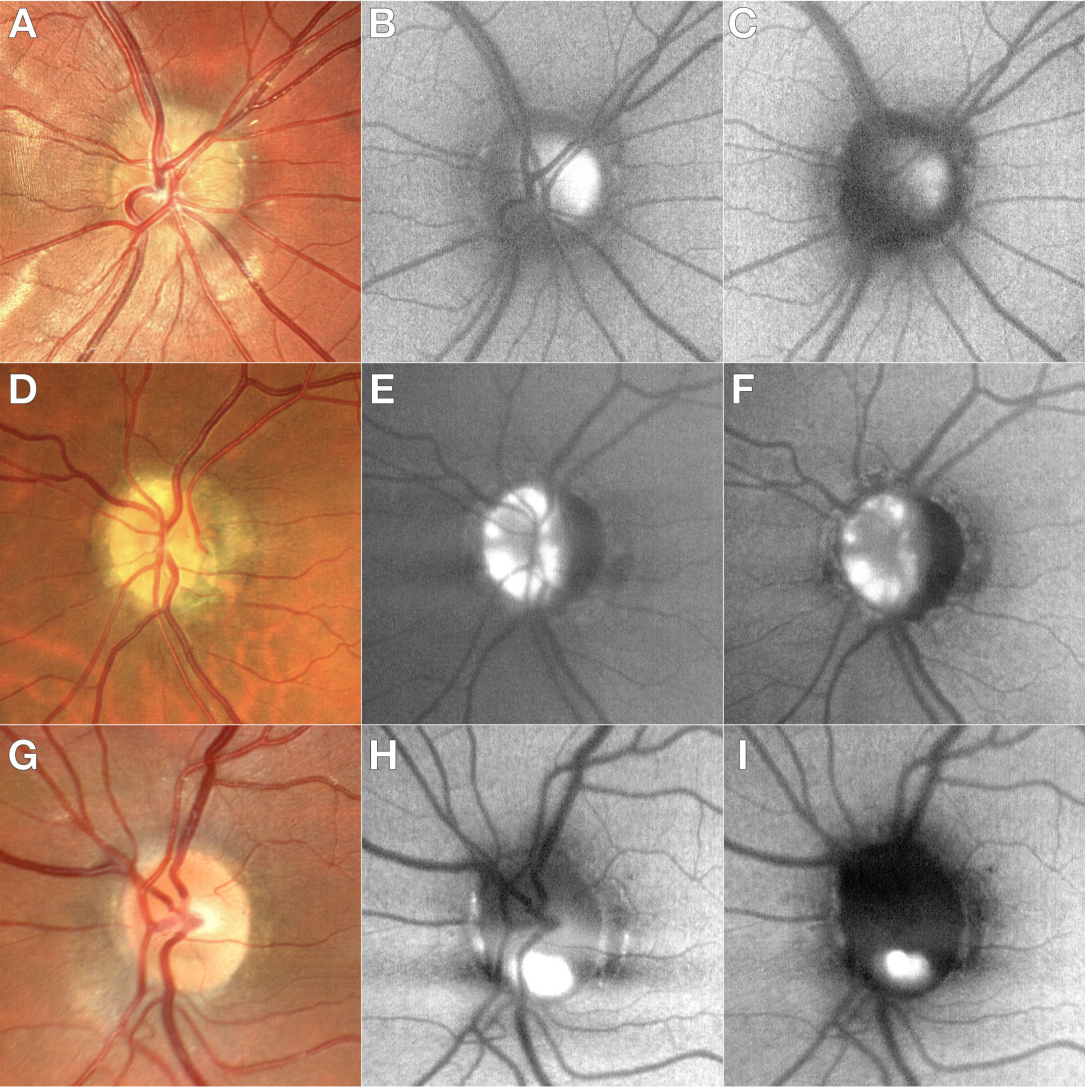
Figure 1: A, D, G: true color imaging. D, E, H: FAF-Blue. C, F, I: FAF-Green. On FAF-Blue, the ONHD reveal a wider hyper-AF area with blurry margins, while the FAF-Green highlight smaller and localized areas of hyper-AF with well-defined margins that are well-contrasted with the adjacent areas of hypo-AF of the optic nerve.

We have recently observed a noticeable disparity in the SW-FAF imaging of ONHD when comparing FAF-Blue and FAF-Green assessed with BLFI technology (ZEISS CLARUS 700, Carl Zeiss Meditec, Dublin, California, USA). The FAF-Blue images reveal a wider hyper-AF area with heterogeneous signal and blurry margins, while the FAF-Green highlight smaller and localized areas of hyper-AF that are well-contrasted with the adjacent areas of hypo-AF of the optic nerve (Figure 1 (Fig. 1)). We suspect that this phenomenon occurs due to the absorption of light by collagens in the optic nerve with the FAF-Blue illumination, with subsequent emission of fluorescence within the detection range (446 nm), creating the impression of a more prominent lesion. As the FAF-Green has deeper penetration, and the interference from the optic nerve collagens is negligible, the margins of the lesions appear more well-defined, and therefore FAF-Green is able to establish more reliably the size of the ONHD, in addition to more accurately detecting deeper drusen.

To the best of our knowledge, this is the first report that brings to light a new rationale in SW-FAF image acquisition of ONHD by comparing FAF-Blue and FAF-Green assessed with the BLFI technology. Even at very close wavelengths, both scan types present their particularities and utilities. Whereas FAF-Blue may have better utility in detecting superficial lesions, the FAF-Green outperformed FAF-Blue in defining ONHD.

## Notes

### Ethics statement

All patients have read and signed the free and informed consent form.

### Competing interests

The authors declare that they have no competing interests.
